# Prevalence and utilization of company integration management in Germany: Results of the 2018 BiBB/BAuA survey of employed persons

**DOI:** 10.1002/1348-9585.12276

**Published:** 2021-09-09

**Authors:** Alfons Hollederer

**Affiliations:** ^1^ The Faculty of Human Sciences (FB 01) University of Kassel Kassel Germany

**Keywords:** disabled persons, job satisfaction, return to work, sick leave, workplace

## Abstract

**Objectives:**

This secondary analysis aims to investigate the implementation of the legally required company integration management (“BEM”) in case of an incapacity for work of at least six weeks and to identify predictors.

**Methods:**

Database is the representative randomized 2018 BIBB/BAuA Employment Survey of 20 012 employed persons in Germany.

**Results:**

Of the 1367 employees entitled to company integration management, 40% received an offer from their employer and 27% accepted it. In the public sector, half of those who were entitled reported an offer. Among those entitled to company integration management, employees under the age of 30, at risk of dismissal, or with fixed‐term employment contract received an offer particularly rarely. Entitled employees with disabilities or in companies with works/staff councils received disproportionately often an offer of company integration management. Logistic regression analyses reveal strong associations between company integration management offer and the duration of incapacity to work. The probability of receiving an offer is almost halved for those entitled in medium‐sized compared to small companies. The higher the level of educational qualification, the higher are odds ratios for an offer. In companies in which employees were less or not satisfied with their work overall, the chance of a company integration management offer is significantly reduced almost by half. The chance of an offer is more than three times higher in companies with workplace health promotion compared to those without.

**Conclusions:**

Only a minority of eligible employees received an offer that is closely associated with health‐promoting corporate culture and job satisfaction.

## 1 INTRODUCTION

With the impacts of demographic change and the increase of statutory retirement age, it is becoming even more important for companies, employees and society to promote the health of employees, prevent illness, rehabilitate and integrate employees with health impairments and disabilities.

In Germany, the rate of sick leave of employed compulsory members of the statutory health insurance funds was 4.25% on average in 2018, and it has increased slightly in recent years.^1^ Statistics on the incapacity to work show for 2018,^1^ that in Germany 95.8% of the 42 million registered cases of incapacity to work ended within a period of 42 days (excluding pensioners). This period also marks the end of continued payment of salaries by employers in case of incapacity to work. However, the remaining few 4.2% of cases cause almost half of the 574 million days of incapacity to work in Germany (48.9%). Incapacity for work places a burden on companies through continued payment of wages in the event of illness and loss of production.

These are important reasons for workplace interventions. Internationally, there is a moderate‐quality evidence that workplace interventions can help employees return to work and reduce the duration of sickness absence.^2^ Disability management has been shown to be effective and efficient for ensuring job retention and occupational reintegration.^3^

In Germany, the legislator therefore reacted as early as 2004 by obliging all employers to implement a special program “company integration management” (“Betriebliches Eingliederungsmanagement [BEM]”). Section 167 (2) of the Ninth Book of the Social Code (SGB IX) stipulates that if employees are continuously or repeatedly unfit for work for more than 6 weeks within 1 year, the employer shall clarify with the relevant representation of interests, with the consent and participation of the person concerned, the options for overcoming the incapacity for work as far as possible and with which benefits or assistance renewed inability to work can be prevented and the job can be maintained. If necessary, the company doctor will be consulted. The person concerned or the legal representative shall be informed in advance of the aims of the company integration management and of the type and scope of the data collected and used for this purpose. If benefits for participation or accompanying assistance in working life come into consideration, the rehabilitation providers or, in the case of severely disabled employees, the Integration Office shall be consulted by the employer. The responsible interest group can request clarification. In the case of severely disabled persons, the representative council for severely disabled persons is also involved. They shall ensure that the employer fulfills the obligations incumbent upon it under this provision.

The responsibility for the implementation of the company integration management thus remains with the employer. The procedure and content of the company integration management are not further regulated by the law. The company integration management is designed as a process and the employee’s participation is voluntary. The procedural processes for individual cases are specified differently in practice.^4^, ^5^, ^6^, ^7^, ^8^ Medical or vocational rehabilitation measures may also be initialized. Usually the process chain would begin with the determination of incapacity for work of more than six weeks in the company. This would be followed by an initial contact with the affected employee and the conduct of an initial interview to clarify the willingness to cooperate. The workplace and the requirements would then be analyzed together with the employee concerned. In the framework of the company integration management, coordination and networking are important components. After the discussion of the case, the measures of company integration management would be specified and concrete measures would be implemented. Operational measures include, for example, reducing the scope of work or working hours and making working time more flexible. Effectiveness monitoring is part of the process. If the integration is evaluated positively, the company integration management is concluded.

The statutory introduction of company integration management aimed at preventing severe disability, loss of earnings, early retirement as well as renewed incapacity for work. This is in line with occupational safety and workplace health promotion.^9^ In Germany, workplace health promotion was established in the 1990s as a voluntary benefit by the statutory health insurance funds and has been continuously expanded since then.^10^, ^11^

In Germany, the implementation of company integration management in companies has been slow despite legal requirements, but there is a lack of representative studies.^12^ The quality of company integration management varies greatly in practice. Particularly, in small‐ and medium‐sized companies, obstacles such as information deficits, lack of prioritization, limited opportunities for integration or illness as a taboo subject have been identified.^13^ Companies report problems in implementing company integration management due to a lack of suitable workplaces in the company.^14^ A nationwide survey of 630 companies by Niehaus et al.^15^ found that 55% of large companies, 38% of medium‐sized companies and 23% of small companies had addressed company integration management in 2006–2007. Only two‐thirds of the companies had an office that monitored work incapacities in relation to the six‐week period. Shortly thereafter, in the European Survey of Enterprises on New and Emerging Risks 65% of firms (with 20 or more employees) report that they take measures to support employees’ return to work following a long‐term sickness absence.^16^ The spread grew with the size of the company. A survey of works councils found that 77% of companies with 20 or more employees offered a company integration management in 2015.^17^ Expert interviews by Ramm et al.^18^ confirmed the low level of awareness of company integration management in small and medium‐sized enterprises.

Loerbroks et al.^19^ recently examined company integration management in Germany only in a cohort study of individuals aged 40–54 who received sickness benefits in 2012. Thirty‐four percent of respondents indicated that they had been offered company integration management at some point until 2015. Seventy‐seven percent of them had accepted this offer. Increasing company size was the strongest predictor of a future company integration management offer. The likelihood of both an offer and acceptance of company integration management was increased among participants affected by mental illness or cancer in 2013.

Overall, there is an empirical research gap on the prevalence and utilization of the legally required company integration management. The aim of the secondary analysis is to investigate the implementation of company integration management in case of an incapacity to work of at least 6 weeks. It aims to identify predictors and analyze the degree to which vulnerable groups are reached.

## 2 METHODS

These analyses were performed by the use of data collected in the framework of the “BIBB/BAuA Employment Survey of the Working Population on Qualification and Working Conditions in Germany 2018” collected by the Federal Institute for Vocational Education and Training (BiBB) and the Federal Institute for Occupational Safety and Health (BAuA) from October 2017 to April 2018.^20^ Data were accessed via a Scientific Use File from the Research Data Center at BiBB (BIBB‐FDZ). The population of the representative survey consists of employed persons in Germany who are at least 15 years old and are engaged in paid work of at least 10 h per week. The random sample comprises 20 012 employees interviewed via computer‐assisted telephone interviews. For extrapolation, the data include adjustment weights (region, household size, occupational status, gender, nationality, education, age). The survey method was described in detail by Rohrbach‐Schmidt and Hall.^21^ The data set contains differentiated information on employed persons and their jobs in Germany. There are already initial basic frequencies of the sample by Lück et al.^22^ and by the BAuA, that published a fact sheet on workplace integration management.^23^ However, both analyses resulted primarily in descriptive reports. The focus of the present analyses is on the prevalence and utilization of company integration management offers since there are research gaps.

The company integration management offer and utilization were surveyed as follows:

1. Due to your longer sick leave, was your employer offering you company integration management, e.g. a reduction in the amount of work, a working time reduction or flexibilization? (Yes/No)
2. Did you accept the offer? (Yes/No)

The purpose of company integration management is to rehabilitate, maintain, and promote the employability of employees who have been incapable for work continuously or repeatedly for more than 6 weeks within the last 12 months.

Associations between company integration management and the following items are examined:

‐ socio‐demographic characteristics (gender, age, highest level of occupational certification, nationality, occupational status);
‐ officially recognized disability;
‐ characteristics of the companies (company size, works/staff council, economic sector, workplace health promotion);
‐ job‐related characteristics (fixed‐term employment, risk of dismissal, support, International Standard Classification of Occupations, job satisfaction).

The research approach for secondary analysis uses descriptive statistics, correlation analysis and binary logistic regression analysis. Pearson’s chi‐square tests are used to test difference hypotheses. Phi coefficients are used as correlation measures for nominally scaled variables in the case of alternative variables. *P*‐value < .05 was used to decide a statistically significant association. In logistic regression analyses, the odds ratio (OR) is a measure of how much greater the probability of an event (such as the offer of company integration management) is in the group with certain characteristics compared to the group without these characteristics. The effect coefficient exp(B) was used in evaluating the influencing variable; it indicates the factor by which the OR is multiplied. For predictions, covariates were incorporated into the analysis. The goodness of the model fit was evaluated with the likelihood function. 95%‐confidence intervals are calculated for the ORs. The analyses were performed using IBM/SPSS‐Statistics 26.

## 3 RESULTS

Table 1 shows an overview of the sample in the 2018 BiBB/BAuA survey of employed persons. According to self‐reporting, 1367 of 20 012 successfully interviewed employed persons were entitled to company integration management because (1) they have stayed home sick or called in sick for at least 31 working days in the last 12 months and (2) they are not self‐employed, freelancers, freelance collaborator or assisting family member. Of these, 551 (40%) stated that they were offered company integration management by their employer. Of these, 68% employees also took advantage of the company integration management offer.

**TABLE 1:** Employed persons entitled to company integration management in the 2018 BIBB/BAuA survey

Characteristics	Employed persons		Offer of company integration management	Acceptance company integration management	*P*‐value (for differences between subgroups)	
	*N*	% of column 2 (weighted)	Line‐% (“Yes, offer” to *N*, weighted)	Line‐% (“Yes, acceptance” to N, weighted)	*P*‐value (offer, col. 4)	*P*‐value (acceptance, col. 5)
*(Column 1)*	*(2)*	*(3)*	*(4)*	*(5)*	*(6)*	*(7)*
BIBB/BAuA Employment Survey total	20 012	100				
Among employees entitled to company integration management	1367	7	40	27		
Among employees with offer	551	3		68		
Among employees accepting offer	368	2				
Employees entitled to company integration management (conditional variables)						
Occupational status						
Worker	402	29	38	78	.500	<.01
Salaried employee	889	65	41	67		
Civil servant	71	5	54	46		
Missing (1), not specified (4)	5	0				
Number of sick days in the last 12 months (AM = 85.5; SE = 66.8)						
31–119 working days	1058	77	36	66	<.001	<.001
120–179 working days	147	11	63	70		
180+ working days	163	12	47	78		
Socio‐demographic characteristics of employees entitled to company integration management						
Gender						
Male	685	50	39	68	.501	.762
Female	683	50	41	69		
Age group (AM = 48.1; SE = 11.1)						
≤30 years	137	10	26	64	<.01	.078
31–40	189	14	39	60		
41–50	358	26	39	75		
51–60	526	39	45	70		
61+	157	12	44	59		
Missings	8	1%				
Highest level of occupational certification						
No vocational training certificate or degree	178	13	31	/	<.001	.154
Dual or school‐based vocational training, civil servants in lower or middle level	952	70	40	69		
Advanced further training (master’s or technician’s certification)	83	6	42	/		
University (of applied sciences), civil servants in upper or higher level	146	11	56	60		
Missings	8	1				
Nationality						
German	1262	92	41	68	.163	.127
Foreign citizenship	106	8	34	/		
Disabled employees entitled to company integration management						
Officially confirmed disability						
No	950	69	38	68	<.01	.765
Yes	416	30	47	69		
Missings	2	0				
Characteristics of companies among employees entitled to company integration management						
Company size						
≤49 employees	484	35	40	76	<.001	<.05
50–249 employees	383	28	32	69		
250+ employees	463	34	50	62		
Missings	38	3				
Works/staff council						
Yes	885	65	45	63	<.001	<.001
No	396	29	32	82		
Missings (35), Company size <5 employees (52)	87	6				
Economic sector						
Public service	382	28	50	57	<.001	<.001
Industry	288	21	45	67		
The craft	165	12	34	/		
Trade	170	12	28	/		
Other services	246	18	35	73		
Another area	100	7	40	/		
Missings	16	1				
Workplace health promotion						
No	737	54	27	78	<.001	<.001
Yes	584	43	57	62		
Missings	47	3				
Job‐related characteristics						
Temporary employment relationship (Worker, salaried employee)						
Temporary	111	8	15	/	<.001	/
Permanent	1176	86	42	71		
Missings	80	6				
Risk of dismissal or closing down (without civil servants)						
Very high or high	132	10	25	52	<.001	<.05
Rather low	453	33	37	75		
There is no danger at all	688	50	44	70		
Missings	95	7				
Support for work from line manager when needed						
Frequently	629	46	47	66	<.001	.596
Sometimes	325	24	40	70		
Rarely	251	18	37	73		
Never	154	11	22	68		
Missings	9	1				
The International Standard Classification of Occupations (ISCO) of International Labour Organization						
Major groups	50	4	38	/		
Managers	83	6	57	65	<.001	<.01
Professionals	319	23	50	58		
Technicians and associate professionals	153	11	42	68		
Clerical support workers	229	17	28	/		
Service and sales workers	11	1	/	/		
Skilled agricultural, forestry and fishery workers	210	15	47	80		
Craft and related trades workers	176	13	28	56		
Plant and machine operators, and assemblers	128	9	33	/		
Elementary occupations	8	1	/	/		
Armed forces occupations						

Note

*N* = 1367; minimal differences by rounding up and extrapolation possible. “/” Extrapolated group figures with too low case numbers are not shown; a slash (/) appears instead.

Abbreviations: AM, arithmetic mean; SE, standard error.

Table 1 provides information on disparities in company integration management provision and utilization between the groups according to socio‐demographic characteristics and work‐related variables. The analysis of company integration management provision by employers should be evaluated in the context of utilization, as this also varies significantly across groups.

It informs about a comparison of company integration management with job‐related characteristics. Only a small proportion of employees is affected by a fixed‐term employment or by a risk of dismissal, but they have been offered company integration management to an extraordinarily small extent. Only 15% of the entitled blue‐ and white‐collar workers in a fixed‐term employment reported that they received an offer of company integration management. Those in that group, who consider themselves at high or very high risk of dismissal, only 25% received an offer of company integration management. In addition, their utilization rate is particularly low with 52%.

Works/staff councils can support company integration management. Entitled persons who worked in companies with a works/staff council received an offer of company integration management significantly more frequently than the other entitled persons in companies that could have had employee representation (prerequisite at least five employees) but did not (45% vs. 32%). However, their take‐up rate of 63% was significantly lower than that of entitled employees without works/staff councils (82%).

**FIGURE 1:**
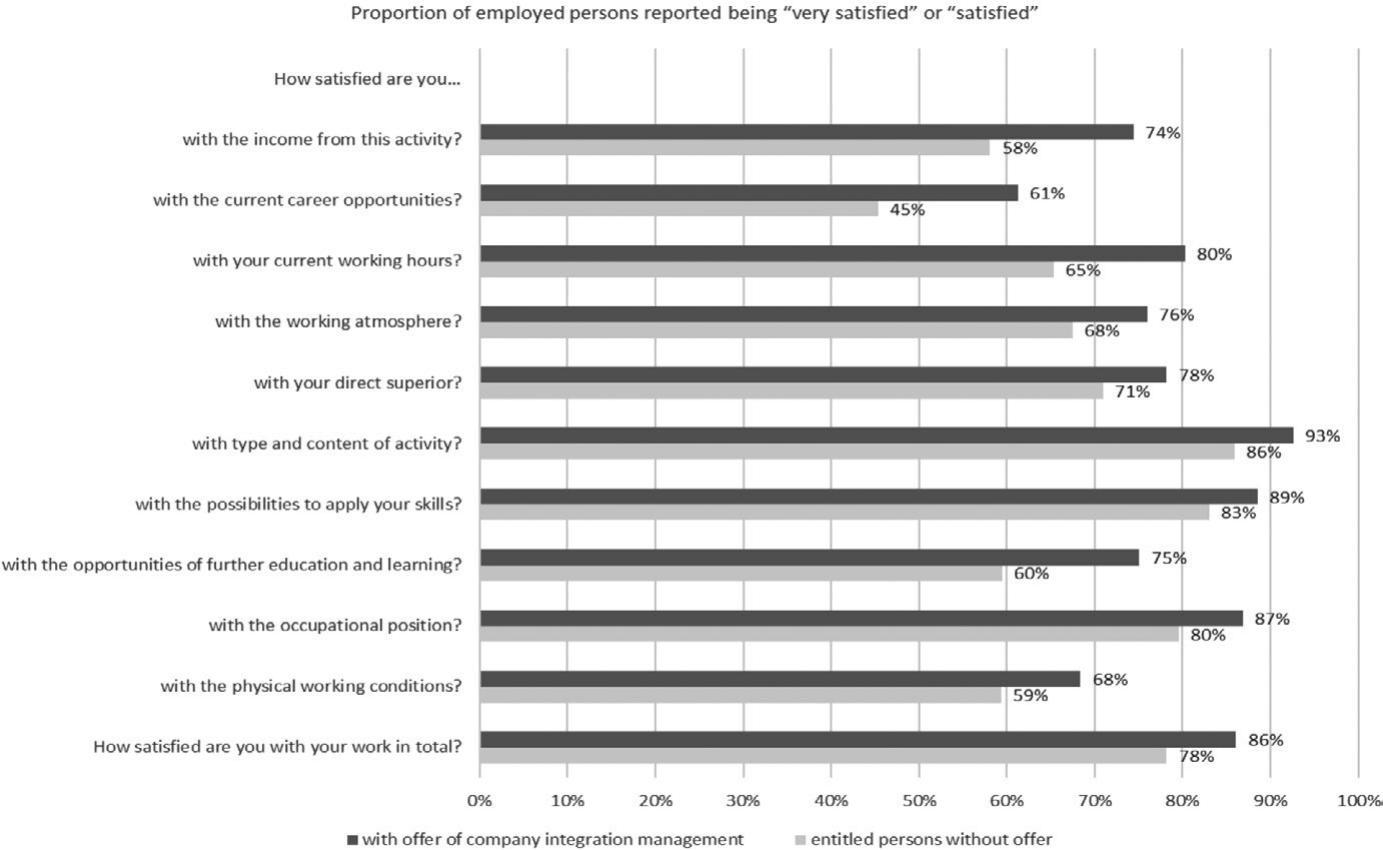
Company integration management offer and job satisfaction in the 2018 BIBB/BAuA survey. Notes: (i) *N* = 1367. (ii) Dichotomized answers to the question “Please tell me now for different aspects of your activity whether you are very satisfied, satisfied, less satisfied or not satisfied with it.” (iii) The both groups of the figure differ in all items in a statistically significant way. *Source*: BIBB/BAuA Employment Survey of the Working Population on Qualification and Working Conditions in Germany 2018; weighted data

An offer of company integration management by the employer goes hand in hand with a high job satisfaction among employees entitled. To the question “And now, all in all: How satisfied are you with your work in total?”, 25% of employees received an offer of company integration management answered “very satisfied” and 61% “satisfied” (entitled persons without offer: 15% and 64%). However, this association applies not only to satisfaction with work overall, but to all queried aspects of the job across a broad spectrum. As shown in Figure 1, this also includes areas that can be addressed by the company integration management, such as working hours, the type and content of the job or physical working conditions. It should be remarked that the associations found only apply to the company integration management offers. All analogously tested associations between the various aspects of job satisfaction and utilization were not statistically significant.

### 3.1 Logistic regression model for offer of company integration management

The binary logistic regression analysis in Table 2 focuses on factors predicting the probability of receiving an offer for company integration management among entitled employees. It takes into account socio‐demographic characteristics, sick days (at least 31 working days), disability, company characteristics and job satisfaction as covariates.

**TABLE 2:** Logistic regression model for offer of company integration management in the 2018 BIBB/BAuA survey

Characteristics	Odds ratio (95%‐CI)
Employees entitled to company integration management (conditional variables)	
Occupational status	
Worker	Ref.
Salaried employee	1.09 (0.80–1.50)
Civil servant	1.27 (0.67–2.42)
Number of sick days in the last 12 months	
31–119 working days	Ref.
120–179 working days	4.51 (2.92–6.96)***
180+ working days	2.39 (1.63–3.51)***
Socio‐demographic characteristics of employees entitled to company integration management	
Gender	
Male	Ref.
Female	1.11 (0.84–1.46)
Age (in years)	1.02 (1.00–1.03)*
Highest level of occupational certification	
No vocational training certificate or degree	Ref.
Dual or school‐based vocational training, civil servants in lower or middle level	1.12 (0.75–1.69)
Advanced further training (master’s or technician’s certification)	1.24 (0.66–2.32)
University (of applied sciences), civil servants in upper or higher level	1.79 (1.04–3.08)*
Nationality	
German	Ref.
Foreign citizenship	0.82 (0.51–1.32)
Disabled employees entitled to company integration management	
Officially confirmed disability	
No	Ref.
Yes	1.25 (0.94–1.66)
Characteristics of companies among employees entitled to company integration management	
Company size	
≤49 employees	Ref.
50–249 employees	0.55 (0.40–0.77)***
250+ employees	0.90 (0.64–1.26)
Economic sector	
Public service	Ref.
Industry	0.97 (0.65–1.44)
The craft	0.65 (0.40–1.07)
Trade	0.71 (0.45–1.12)
Other services	0.78 (0.53–1.15)
Another area	1.04 (0.61–1.77)
Workplace health promotion	
No	Ref.
Yes	3.16 (2.41–4.15)***
Satisfaction of employees entitled to company integration management	
General job satisfaction	
Very satisfied or satisfied	Ref.
Less satisfied or not satisfied	0.54 (0.39–0.76)***
Pseudo‐*R* ^2^ (Nagelkerkes)	0.219
−2 Log‐Likelihood	1477.064
*N*	*1254*

Note

Weighted findings.

Abbreviation: CI, confidence interval.

* *P* <.05.

*** *P* <.001.

The company integration management offer is associated with the duration of incapacity for work, which was recorded in the survey with the number of sick days at work in the last 12 months (Table 1). According to the interview instructions, 1 month corresponds to 20 working days. The logistic regression model confirms, that the number of sick days in the last 12 months turns out to be a very important influencing factor. The sick days are grouped into three categories. Contrary to expectations, the highest probability of a company integration management offer lies in the middle category with a summarized incapacity to work of 120–179 working days with a significantly increased OR of 4.51. Apparently, despite legal entitlement from 6 weeks of incapacity to work, a company integration management offer is often only made later with significantly longer incapacity to work. In contrast, serious illnesses with very long periods of incapacity to work of at least 180 working days correspond to an OR of 2.39.

The characteristics of the company and the workplace were identified as particularly important influencing variables. Contrary to expectations, only 32% of those entitled from medium‐sized companies received an offer of company integration management, which was used by 69%. In contrast, both rates are higher among those entitled to company integration management in small companies. In medium‐sized companies with 50–249 employees, the probability of a company integration management offer is significantly reduced by a factor of 0.55 in relation to small companies in the logistic regression model.

There is a strong correlation between company integration management and workplace health promotion (Table 1). Entitled employees working in a company with health promotion measures in the last 2 years were offered company integration management significantly more often than the other employees entitled (57% vs. 27%). In the multivariable cross‐sectional analysis, the presence of workplace health promotion is closely associated with a three times higher chance of an offer of company integration management (OR = 3.16).

In companies where the employees entitled are less satisfied or not satisfied with their work overall, the probability of a company integration management offer is almost halved in a statistically remarkable way in the logistic regression model (OR = 0.54).

In terms of economic sectors, company integration management is most widespread in the public sector (Table 1). In this logistic regression model, the probability of receiving a company integration management offer also varies somewhat according to the economic sector of the company.

Socio‐demographic variables have only a relatively small influence on the prediction of a company integration management offer. The multivariable analysis confirms a small influence of age on the receipt of a company integration management offer.

Among those entitled without a vocational qualification, there are relatively few company integration management offers (Table 1). The rates of company integration management offer increase with the level of the highest educational qualification. Higher level of occupational certification improves the likelihood of a company integration management offer, especially for graduates of a university of applied sciences, university, or for civil servants in upper or higher level (OR = 1.79).

Almost one‐third of those entitled have an officially recognized disability (Table 1). They disproportionately often reported receiving a company integration management offer. An officially recognized disability of the beneficiaries is associated with a slightly increased OR of 1.25 for a company integration management offer.

## 4 DISCUSSION

The 2018 BIBB/BAuA survey shows that, despite legal requirements since 2004, company integration management has only been implemented in a minority of companies. Company integration management is offered to 40% of employees, of whom around two‐thirds take advantage of it. All in all, this magnitude is very much in line with the earlier studies presented above, which used other data sources with different restrictions.^15^, ^19^ There remains a great need for research on the reasons for non‐utilization because the reasons for refusal were not captured. In accordance with Loerbroks et al.^19^ in the present evaluation the acceptance of offers for company integration management is particularly high in small companies. In addition, utilization correlates with other company characteristics such as works/staff council, economic sector, workplace health promotion and risk of dismissal. Since the consent of the affected person is required for company integration management, mutual trust and reliability are needed. Employers usually do not know the diagnosis. Possibly, from the employees’ point of view, the protection of the data is a reason for not making use of the data if they fear dismissal due to illness. In addition, not all long‐term illnesses are known to require company integration management, for example in case of a bone fracture.

The binary logistic regression analyses aimed to identify the determinants of offers of company integration management. Socio‐demographic characteristics, health, and work‐related variables were used as predicting factors. The association between offer of company integration management and durations of work incapacity suggests that a large proportion of offers of company integration management starts with a time lag. This could be due to administrative reasons because of the determination of the six‐week period, especially in cases of multiple incapacities to work. It may also be related to the personal presence in the company directly after a convalescence.

A strong predictor for a company integration management offer is the performance of health promotion measures in the company. This is consistent with observations by Ramm et al.^18^ Workplace integration management is apparently better integrated into an existing culture of prevention in the company and into a holistic workplace health management system.

The probability of a company integration management offers increases significantly with job satisfaction. Job satisfaction is a key indicator for many conditions in the company and in the workplace. However, the offer of company integration management by employers not only correlates positively with the general job satisfaction of the employed, it is also associated with higher satisfaction in all individual aspects surveyed. This is also true for the satisfaction with the direct superior, who may be involved in the company integration management, or the satisfaction with working atmosphere. The significance of working atmosphere is in congruence with the expert interviews by Ramm et al.^18^ in which both employers and employees confirmed that the working atmosphere is very important.

The proportion of employees in small companies who reported that they received an offer of company integration management was surprisingly high. This was unexpected, keeping in mind the results of the studies mentioned above.^13^, ^15^, ^18^, ^19^ Smaller companies lack elaborate structures for holistic health management, but they have other advantages that may have had a positive impact here: there is usually a good flow of communication, information is easily accessible, the hierarchy is flat, and there is a close social relationship between the company director and the staff.^24^

In the multivariable analyses, employees with the highest level of education at a university of applied sciences, university, university of cooperative education or as higher civil servants have a greater chance of receiving a company integration management offer. It is also possible that company offer strategies are influenced by criteria such as replaceability in times of a shortage of skilled workers or cost‐benefit ratios.

There is a need for further research not only on the utilization of company integration management, but also on the quality and results of the process. There is no nationwide overview of which measures have been agreed upon and whether they have had an effect from the perspective of the employees. It would also be important to know who has assumed responsibility for company integration management in the company (e.g. human resources department, direct supervisor, works council) and which internal actors (company doctor, representative council for the severely disabled) and external network partners (integration offices, integration specialist services, social insurance funds) were involved.

It should be noted that the proportion of those entitled to company integration management who have received an offer is slightly lower than the proportion of reported workplace health promotion in the last 2 years. However, the difference lies in the fact that company integration management is a mandatory statutory duty of the employer. In terms of the principle of equal treatment, therefore, post‐regulation or tax incentives are recommended. It is noteworthy that even in the public sector and among civil servants, only around half of the employees entitled to company integration management report an offer. More effort would be worthwhile, both humanly and financially. Economic cost‐benefit analyses suggested a cost‐effectiveness of company integration management at a ratio of 1 to 4.81 in savings.^25^

As a methodological limitation, it should be pointed out that representative sample surveys are in principle subject to random errors. The smaller the number of cases, the more statistically uncertain the results. For this reason, multivariable evaluations of the utilization by those entitled to company integration management were not performed. In principle, only the perception of the interviewees can be investigated by means of their self‐reports. The validity of the data also depends on memory, as in the case of sick days, and knowledge, such as the exact size of the company. An “omitted variable bias” cannot be excluded in the multivariable regression analyses. It would be desirable to ask about the diagnoses of incapacity to work and the above‐mentioned aspects of the process of company integration management in future waves of the BiBB‐/BAuA‐employee survey 2018.

## 5 CONCLUSION

The 2018 BiBB/BAuA survey of employees shows interesting results of company integration management between aspiration and reality: Only a minority of entitled employees received a company integration management offer, which is then accepted by the majority. The chance of receiving an offer is closely associated with company characteristics, health‐promoting corporate culture, job satisfaction, and the highest professional qualifications. There is a need for implementation research, development and regulation to ensure that the legal requirements are implemented in all companies.

## CONFLICT OF INTEREST

The author declares that there is no conflict of interest.

## DISCLOSURE

*Ethical approval:* N/A. *Informed consent:* N/A (Scientific Use File). *Registry and the Registration No. of the study/Trial:* N/A. *Approval of the research protocol:* N/A. *Animal Studies:* N/A.

## Data Availability

The data that support the findings of this study are openly available in Data Research Centre at the Federal Institute for Vocational Training and Education (BIBB‐FDZ) at http://doi.org/10.7803/501.1 8.1.1.10, reference number (SUF) ZA7574.^20^
